# Tooth oxygen isotopes reveal Late Bronze Age origin of Mediterranean fish aquaculture and trade

**DOI:** 10.1038/s41598-018-32468-1

**Published:** 2018-09-20

**Authors:** Sisma-Ventura Guy, Tütken Thomas, Zohar Irit, Pack Andreas, Sivan Dorit, Lernau Omri, Gilboa Ayelet, Bar-Oz Guy

**Affiliations:** 1 Israel Oceanographic & Limnological Research, Haifa, Israel; 2 Institute for Geosciences, Johannes-Gutenberg University of Mainz, Mainz, Germany; 3 Department of Isotope Geology, Georg-August-University of Göttingen, Göttingen, Germany; 4 Oranim Academic College, Kiryat Tivon, Israel; 5 Zinman Institute of Archaeology, University of Haifa, Haifa, Israel; 6 Department of Maritime Civilizations, Charney School of Marine Sciences, Haifa, Israel; 7 The Leon Recanati Institute for Maritime Studies, University of Haifa, Haifa, Israel; 8 The Department of Archaeology, University of Haifa, Haifa, Israel

## Abstract

Past fish provenance, exploitation and trade patterns were studied by analyzing phosphate oxygen isotope compositions (δ^18^O_PO4_) of gilthead seabream (*Sparus aurata*) tooth enameloid from archaeological sites across the southern Levant, spanning the entire Holocene. We report the earliest evidence for extensive fish exploitation from the hypersaline Bardawil lagoon on Egypt’s northern Sinai coast, as indicated by distinctively high δ^18^O_PO4_ values, which became abundant in the southern Levant, both along the coast and further inland, at least from the Late Bronze Age (3,550–3,200 BP). A period of global, postglacial sea-level stabilization triggered the formation of the Bardawil lagoon, which was intensively exploited and supported a widespread fish trade. This represents the earliest roots of marine proto-aquaculture in Late Holocene coastal domains of the Mediterranean. We demonstrate the potential of large-scale δ^18^O_PO4_ analysis of fish teeth to reveal cultural phenomena in antiquity, providing unprecedented insights into past trade patterns.

## Introduction

Fishing was an essential economic component of many ancient societies, as evidenced by the presence of fish remains, fishing gears, and fish-associated artifacts in numerous archaeological sites world-wide^1–5^. In the southern Levant, past exploitation and trade of fish has been explored primarily based on the occurrences of fish bones in coastal, riverine and lake-side archaeological sites and through inference from the modern distribution patterns, habitat preferences and ecological niches of these fish species. In the Levant, this has mostly been done for fish that a priori were identified as ‘exotic’. For example, the identification of key Nilotic species such as *Lates niloticus* (Nile perch) and *Bagrus* sp. (Bagrid catfish) in archeological sites of the southern Levant testified that long-range trade systems between Egypt and Canaan have emerged more than 5000 years ago (during the Early Bronze Age)^6–8^^.^

The gilthead seabream (*Sparus aurata*, Linnaeus, 1758) frequently appears in archaeological sites of the southern Levant, since prehistoric times (Late Pleistocene)^4–6^. This species is characterised by thick-enamelled, molar-like teeth (Fig. S1), which are used for cracking shellfish (i.e., bivalves, gastropods and crustaceans)^9,10^. *Sparus aurata* is an euryhaline and eurytherm marine fish which migrates between near-shore, inshore (lagoons) and open sea environments^11–13^. Thus, while the appearance of *S*. *aurata* in inland sites clearly indicates long range trade systems^6,7^, remains of this species in Levantine coastal sites have so far been interpreted as reflecting local fishing activity^6–8^.

State of the art research methodologies provide multiple empirical ways to explore trade and maritime connections of desirable fish source marketing to distant places. For example, past provenance and long-range trade of fish from the North Atlantic have been studied using the C and N stable isotopes of bone collagen (Atlantic cod)^14–18^, and by aDNA analysis^18,19^. However, fish bone C and N isotope analyses require the preservation of collagen, and they are limited to “young” fish because constant bone remodeling causes the isotopic signature to adjust to local conditions in adult fish^14,15^. In the North Aegean (northeast Mediterranean), these analyses showed no clustering with locality or species, and for both isotopes they demonstrated a general overlap between freshwater and marine fish, probably due to bone diagenesis^20^. Hence other isotopic proxies are required to assess fish provenance.

**Figure 1:**
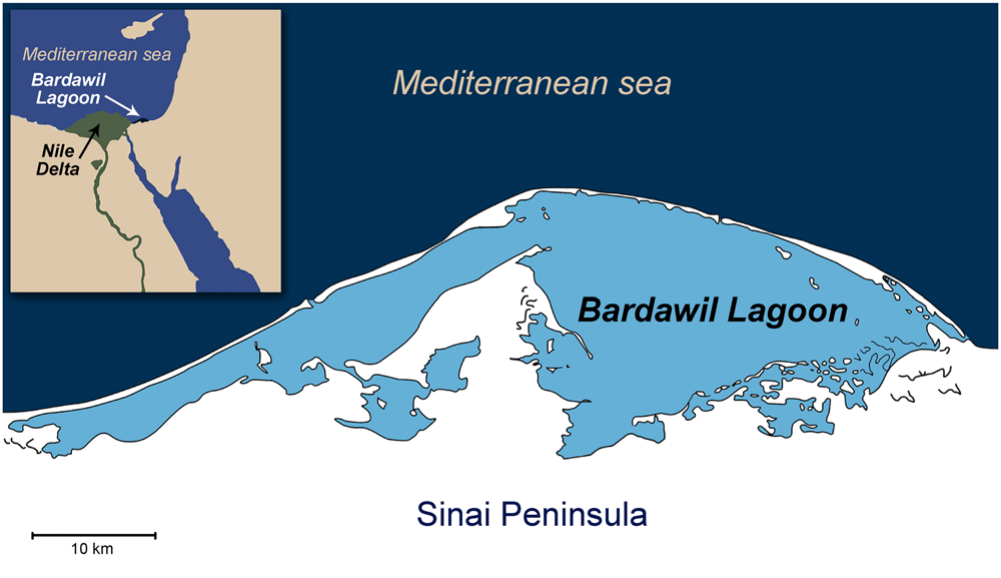
A map of the Bardawil Lagoon of the northern Sinai coast – the main nursery of *Sparus aurata* in the southeast Mediterranean basin.

Longinelli and Nuti’s^21^ pioneering study on the distribution of the phosphate oxygen isotopes (δ^18^O_PO4_) in fish bioapatite demonstrated that δ^18^O_PO4_ values are controlled by the water temperature and oxygen isotope composition of the ambient water (δ^18^O_Water_)^22,23^. Enameloid of fish teeth is highly resistant to diagenetic alterations^24^, and thus in many cases preserve information regarding the original salinity and temperature^25,26^ of their past aquatic habitat (i.e. marine, rivers, lakes, and lagoons)^27–30^. In closed or semi-closed water bodies with a high degree of evaporation, the δ^18^O_Water_ are enriched in ^18^O relative to the seawater that feeds them^23,31^ (Fig. S2). Enameloid of fish teeth from such water bodies carry distinctively high δ^18^O_PO4_ values, which reflect hypersaline habitats of the fish^23,28^. The Bardawil lagoon (*Sabkhat al Bardawil*; along the northern coast of Sinai, Egypt (Fig. 1, 31°09\N, 33°08\E) is such a water body^23,31^.

This study builds on Sisma-Ventura *et al*.^28^, where phosphate oxygen isotopes of tooth enameloid of *S*. *aurata* were used as a new proxy to identify the provenance of archaeological fish remains from the Iron Age in the south-east Mediterranean. Our aim is to provide a first long-range assessment of the sources, exploitation and trade of *S*. *aurata* in the context of Egypto-Levantine inter-regional interaction and commercialism.

**Figure 2:**
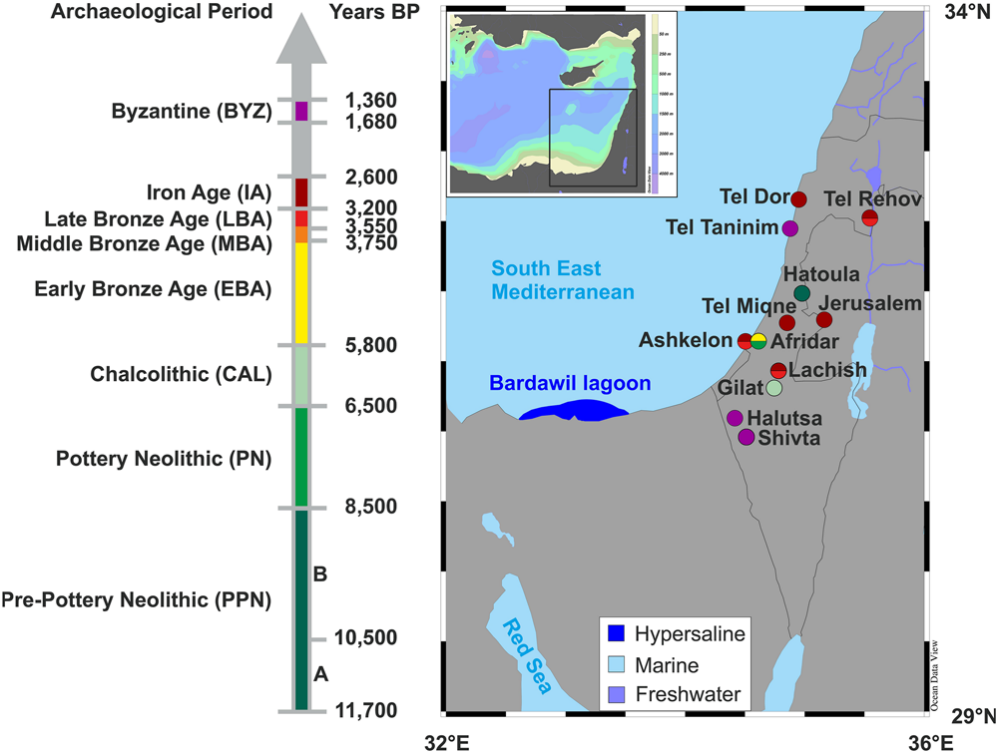
Map of archaeological sites in the southern Levant from the beginning of the Pre-Pottery Neolithic (PPN: ~9,700 years BCE) until the Byzantine period (BYZ: 300 to 600 CE), from which *S*. *aurata* remains were analysed for their phosphate oxygen isotope composition. Also indicated is the location of the hypersaline Bardawil lagoon along the north coast of Sinai, Egypt, which is the proposed source of *S*. *aurata* with high δ^18^O_PO4_ values of >23.5‰.

We assess the provenance of ancient *S*. *aurata* from archaeological layers in the southern Levant by using phosphate oxygen isotope analysis of fish teeth enameloid as a proxy for fish habitat salinity. We analysed the oxygen isotope ratio (^18^O/^16^O in the PO_4_ group, expressed as δ^18^O_PO4_ value; see Methods) of enameloid phosphate of the first molariform teeth (*n* = 100; Table S1) and jawbones (*n* = 24, Table S1) of this species from a broad range of 12 coastal and inland archaeological sites spanning the Pre-Pottery Neolithic to the Islamic period: ~9,700 BCE to 600 CE (~11,700–1,400 years BP; Fig. 2).

During this time span, southern Levantine societies evolved from hunting-gathering, to sedentary ways of lives, to complex societies, to territorial states, and were intermittently subsumed under the aegis of vast or lesser empires – from New Kingdom Egyptians in the Late Bronze Age (c. 1,500–1,200 BCE) to the Muslims of the 7^th^ century CE^32,33^. The fortunes of the Mediterranean southern Levant have always been intertwined with that of its Saharan neighbour in Egypt. Those two different agro-ecological regions were economically interdependent and from the first consolidation of centralized power in Egypt in the 3^rd^ millennium BCE (the Egyptian Old Kingdom), Egypt episodically controlled the southern Levant, whence it could extract the Mediterranean products required for its subsistence and spiritual life. Interconnections between the two regions, however, were not only dictated by cultural and political factors but by climatic fluctuations in both^34,35^. Traffic between Egypt and the Levant was conducted through marine or terrestrial routes through northern Sinai, which was both affected by it in antiquity and provides archaeological proxies for its intensity today. The main question we asked is whether the distribution of these fish to the Levant was a historically-unique and context-dependant phenomenon or rather of more sustainable nature.

## Oxygen Isotopes as Proxy of Fish Aquatic Environment

Bioapatite of fish teeth forms in oxygen isotopic equilibrium with the body fluid at ambient water temperature and δ^18^O_Water_^21–23^. Thus, fish record both the environmental temperature and δ^18^O_Water_ in their bone and tooth δ^18^O_PO4_ values^25–30^. While the δ^18^O_Water_ of the oceans generally shows small variations^36,37^, closed or semi-closed water bodies, such as coastal lagoons, show significantly elevated and variable δ^18^O_Water_ values^23,31^, which are recorded in the hard tissues (i.e. teeth) of migratory fish that exploit these habitats^23,28–30^.

**Figure 3:**
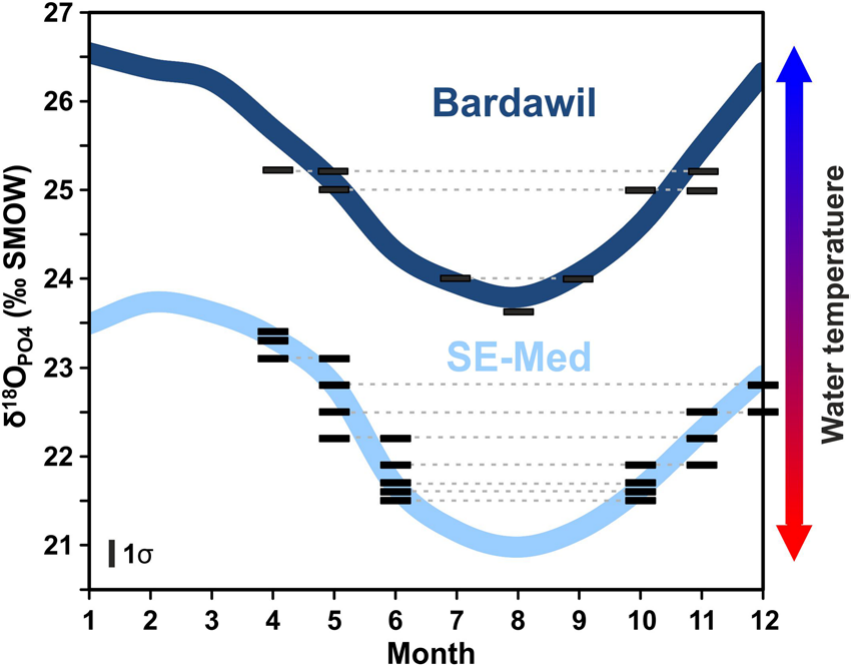
Blue sinusoidal curves represent the seasonal range expected for fish bioapatite δ^18^O_PO4_ values forming in isotope equilibrium with the water of the hypersaline Bardawil lagoon and southeast Mediterranean, respectively. For the Bardawil lagoon an average δ^18^O_SW_ of 3.7‰ and a water temperature range of 14 to 28 °C^23,31,39^ and for the southeast Mediterranean an average δ^18^O_SW_ of 1.6‰ and a water temperature range of 15 to 30 °C^37,38^ were used. Teeth of modern *S*. *aurata* fall within the predicted δ^18^O_PO4_ trend of their according habitat (data for Bardawil fish are from Kolodny *et al*.^23^; data for southeast Mediterranean fish: Sisma-Ventura *et al*.^28^ (this study), reflecting the season of tooth formation. Note that molariform tooth crown mineralisation seems to have occurred year around. This reference frame was used to infer the habitat of ancient *S*. *aurata*.

The temperatures of the Eastern Mediterranean littoral generally range from 15 °C in late winter (February–March) to 30 °C in summer (July–August)^37^. Variations in East Mediterranean δ^18^O_Water_ are small^36–38^, varying between 1.4‰ (February–March) and 1.8‰ (July–August). Therefore, the calculated δ^18^O_PO4_ values (range: 21.2–24.2‰) for bioapatite forming in isotope equilibrium with seawater^22^ (see Methods) in the southeast Mediterranean reflect mostly the seasonal changes in water temperature (Fig. 3). This agrees well with the δ^18^O_PO4_ range from 21.5‰ to 23.4‰ (*n* = 18) in teeth of modern *S*. *aurata* caught in the southeast Mediterranean littoral zone (Fig. 3). In contrast, *S*. *aurata* from the Bardawil lagoon along the Southeast Mediterranean (Sinai) coast display significantly higher δ^18^O_PO4_ values, between 23.5‰ and 25.4‰^23^ (Fig. 3). Bardawil lagoon Sparidae are adapted to a salinity value as high as 60‰^23^. The Bardawil instrumental salinity range typically varies between 36.9‰ and 74.5‰, but could temporarily reach higher values between 70‰ and 90‰, at times when the inlets to the sea were closed artificially^39^.

The Bardawil lagoon (Fig. 1) is a large (30 km long, 14 km max. width), shallow (0.3–3 m deep) hypersaline coastal lagoon, separated from the Mediterranean Sea by a narrow sandbar^39^. The Bardawil is connected to the sea via two small natural inlets (*Boughaz Zaranik*). Water exchange in the lagoon is controlled by Mediterranean Sea tides with a mean height of 50 cm. As a result, it has an elevated salinity level and δ^18^O_Water_ values around 3.7‰ (range: 1.8‰ near the Mediterranean inlet, reflecting inflowing seawater, up to 7.2‰^23,31^ (Fig. S2). The unique environmental conditions of the Bardawil lagoon: shallow, warm (17.3**–**28.3 °C) and hypersaline water (39.0**–**74.5‰) provides optimal growth conditions for several species of fish, including *S*. *aurata*^11^. Today, juveniles of *S*. *aurata* enter the lagoon seeking shelter and food. At about two years of age^11,13^, most fish reach sexual maturity and leave the nursery, migrating back into the open sea where they live in a variety of habitats such as sea grass beds and sandy or rocky bottoms^13,40^.

Fish teeth evolve from the epidermal eruption in the skin of the jaw and are continuously replaced throughout the fish’s life cycle^41^. The δ^18^O_PO4_ values in teeth of modern *S*. *aurata* cover nearly the entire seasonal range of predicted δ^18^O_PO4_values for teeth formed in isotopic equilibrium with the southeast Mediterranean and the Bardawil lagoon water in the according temperature and salinity range, respectively (Fig. 3). We, therefore, assume that tooth formation and replacement occurs on a seasonal basis.

**Figure 4:**
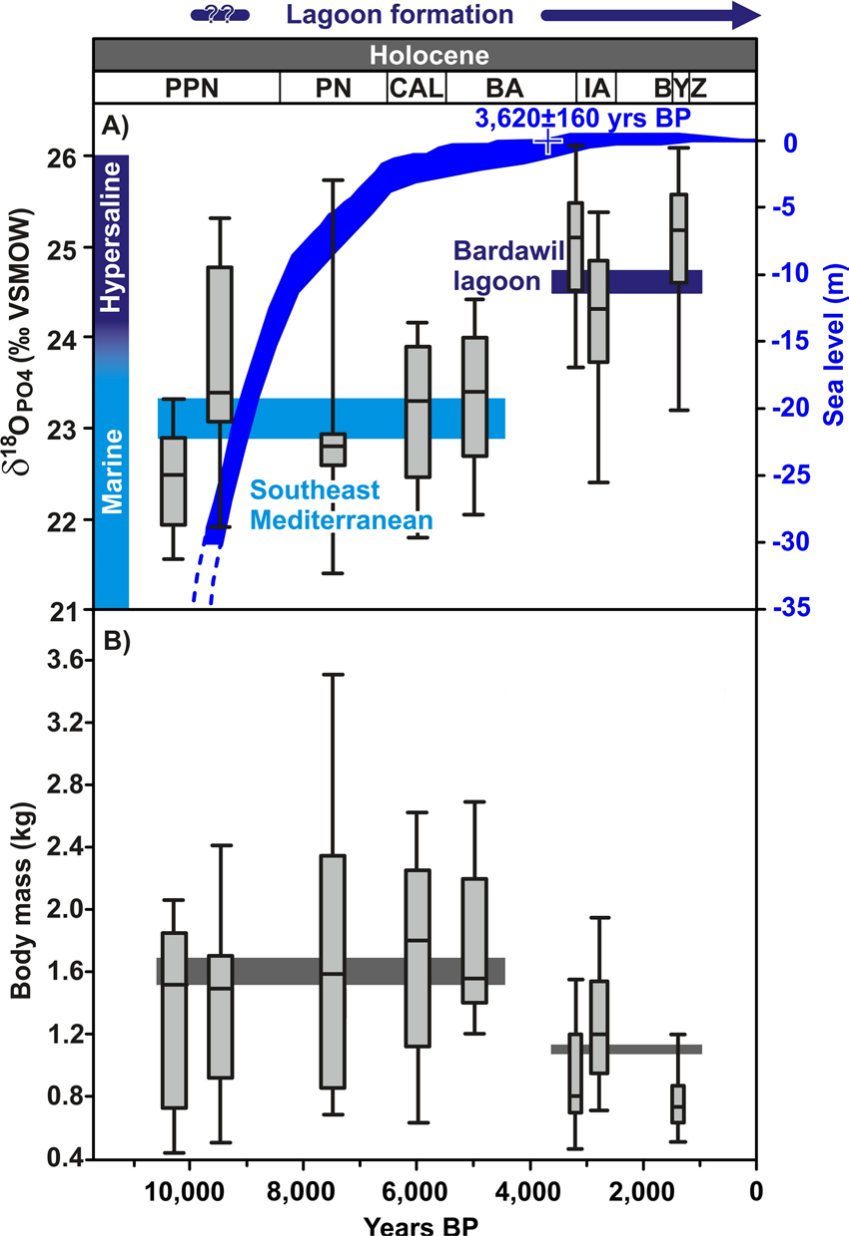
Temporal evolution of phosphate oxygen isotope (δ^18^O_PO4_) composition in archaeological *Sparus aurata* tooth enameloid and estimated body mass. (**A**) Box plots of δ^18^O_PO4_ values of *S*. *aurata* from 12 different archaeological sites in the southern Levant (Fig. 2), spanning the time from the Pre-Pottery Neolithic until the Byzantine period (Table S1). Note, different width of the box plots reflects the age ranges of each site (see Table S1 for details). Changes of Eastern Mediterranean Sea level are taken from^44,45^. The sea level stabilised at the present level (±1 m) about 1,600 years BCE^46^, allowing the formation of the shallow, hypersaline Bardawil lagoon on the shallow shelf of the Sinai coast (Fig. 1). For comparison on the left y-axis the ranges of δ^18^O_PO4_ values expected for *S*. *aurata* that formed their teeth in the Bardawil lagoon and in the southeast Mediterranean (Fig. 3) are given. (**B**) Box plots of estimated body mass of *S*. *aurata*. Tooth δ^18^O_PO4_ of *S*. *aurata* increased to hypersaline values and at the same time fish body mass decreased significantly. Coloured horizontal boxes represent mean values and standard error of δ^18^O_PO4_ and body mass of *S*. *aurata* from the PPN-EBA and LBA-BYZ time intervals.

**Figure 5:**
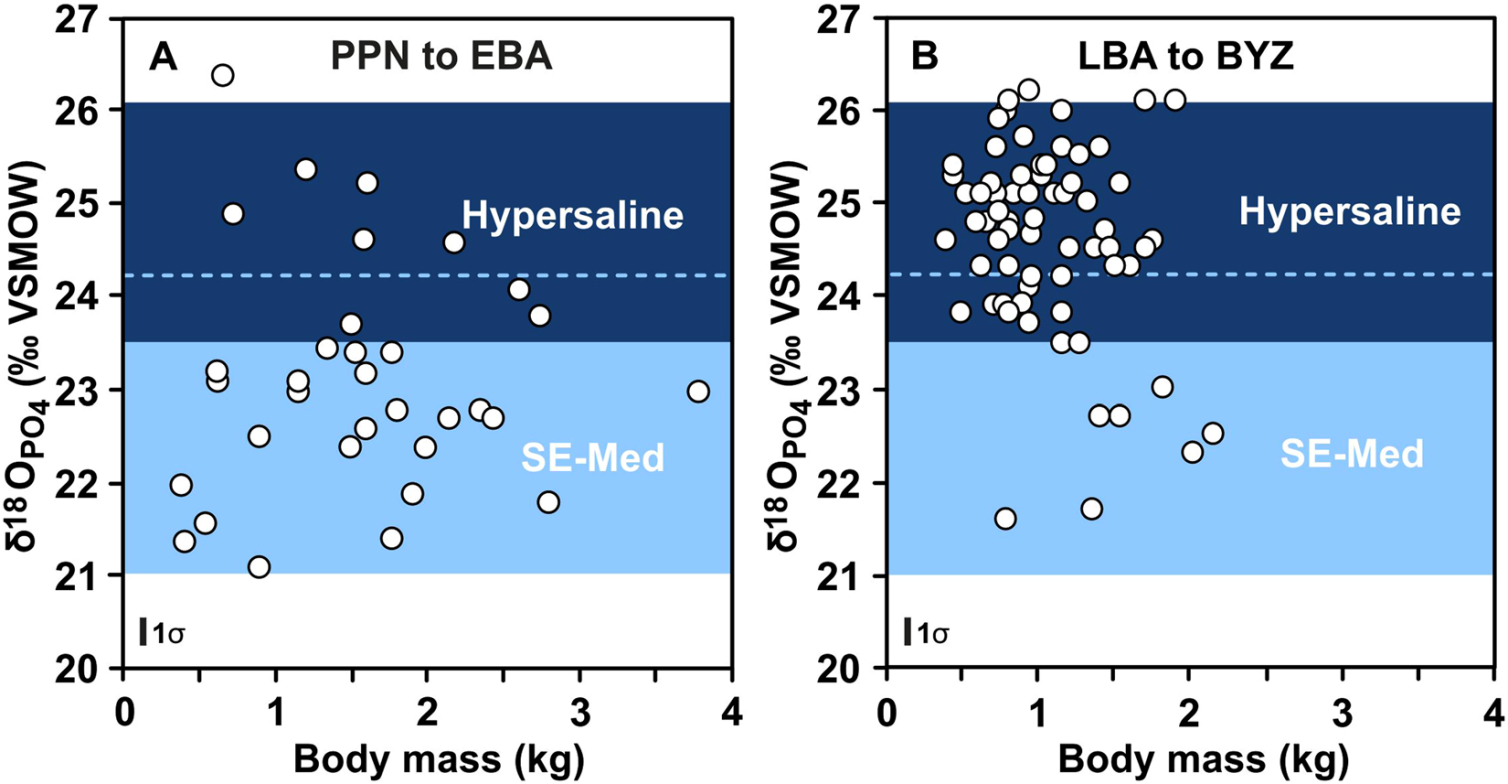
Enameloid phosphate oxygen isotope composition (δ^18^O_PO4_) of *S*. *aurata* teeth versus estimated fish body mass (inferred from molariform tooth size; see text for details) for two different time intervals: (**A**) Pre-Pottery Neolithic (PPN) to Early Bronze Age (EBA): 9,700 to 3,000 BCE. The stippled light blue line marks the lower threshold for unambiguous hypersaline δ^18^O_PO4_ values of *S*. *aurata* from the Bardawil lagoon. Note that before the LBA the few *Sparus* with such high δ^18^O_PO4_ values derive from other, now submerged hypersaline lagoons as the Bardawil lagoon was not present at this time; (**B**) Late Bronze Age (LBA) to Byzantine period (BYZ): 1,200 to 600 years BCE.

The δ^18^O_PO4_ of fish teeth from the archaeological sites in the southern Levant indicate that *S*. *aurata* were caught in two distinct habitats: the southeast Mediterranean littoral characterised by low δ^18^O_PO4_ values (21.5–23.5‰) and a hypersaline environment reflected by higher δ^18^O_PO4_ values (>23.5‰ measured value and >24.2‰ according to the predicted range; see text below for details) (1-Way ANOVA: F = 7.0978, p < 0.001; Table S1, Fig. 4). Thus, it is possible to unambiguously distinguish fish caught from southeast Mediterranean coastal seawater with lower δ^18^O_PO4_ values from those caught in hypersaline lagoonal water, characterised by high δ^18^O_PO4_ values (Table S1; Fig. 5). However, δ^18^O_PO4_ values between 23.5‰ and 24.2‰ can potentially occur in both habitats. Thus, only δ^18^O_PO4_ values exceeding 24.2‰ are considered to reflect unambiguously hypersaline habitats.

Notably, records from sediment cores of the Levant coast, dated to the Holocene, show no evidence for any hypersaline lagoon, similar to the Bardawil, neither in the dimension, nor in the unique environmental setting of this habitat^42,43^. Therefore, as is the case today, the Bardawil lagoon was the only known source for archaeological *S*. *aurata* with hypersaline δ^18^O_PO4_ values consumed in the Levant. A Bardawil provenance for these specimens from the Late Bronze Age (LBA; ~3,200 BP) to the Byzantine period is further supported by the calculated water temperatures, ranging between 16 and 28 °C (using the mean Bardawil δ^18^O_Water_ of 3.7‰), which agree well with the annual water-temperature range of the present-day Bardawil lagoon^39^. We note that the few hypersaline fish dated to the PPN-EBA period (early to mid-Holocene), were likely caught off the southern Levantine shore, and were not exported from the Bardawil, as proposed for the LBA and onward (Late Holocene). This suggests that short-lived, hypersaline lagoons may have formed along the Levant coast when the rapidly rising sea level flooded low-lying coastal areas during the Early Holocene^43,44^.

## Identification of Fishing Grounds/Habitats and Formation of Coastal Lagoons in the Southeast Mediterranean

A key factor in the formation of coastal lagoons in the southeast Mediterranean was the post-glacial stabilisation of the sea level. Over the last 4,000 years sea level stabilised close to its present-day level^44,45^, reaching the current level (±1m) at around 3,620 ± 160 years BP^46^ (1,620 BCE; based on optically stimulated luminescence dating of marine sand deposits, overlain by aeolian sand) (Fig. 4A). Sea-level stabilisation allowed the formation of the perennial shallow hypersaline Bardawil lagoon along the northern Sinai coast, due to the establishment of long-shore currents that transported Nile sands which built up blocking sandbars^42,43^.

During the Early Holocene (Pre-Pottery Neolithic; PPN): PPNA; ~9,700 years BCE; 11,700 years BP) δ^18^O_PO4_ values indicate that *S*. *aurata* was captured mainly from southeast Mediterranean waters and to a lesser extent from hypersaline lagoons (Fig. 4A). The latter results are the first proof of the past existence and exploitation of hypersaline coastal lagoons along the eastern Mediterranean coast during the Early Holocene. Due to the sharp rise in sea level at the onset of the post-glacial period the nature of these lagoons remains unknown. Nevertheless, local fishing in Mediterranean littoral waters was previously assumed based on fish remains from the now-submerged PPNC site of Atlit-Yam in northern Israel^47^.

**Figure 6:**
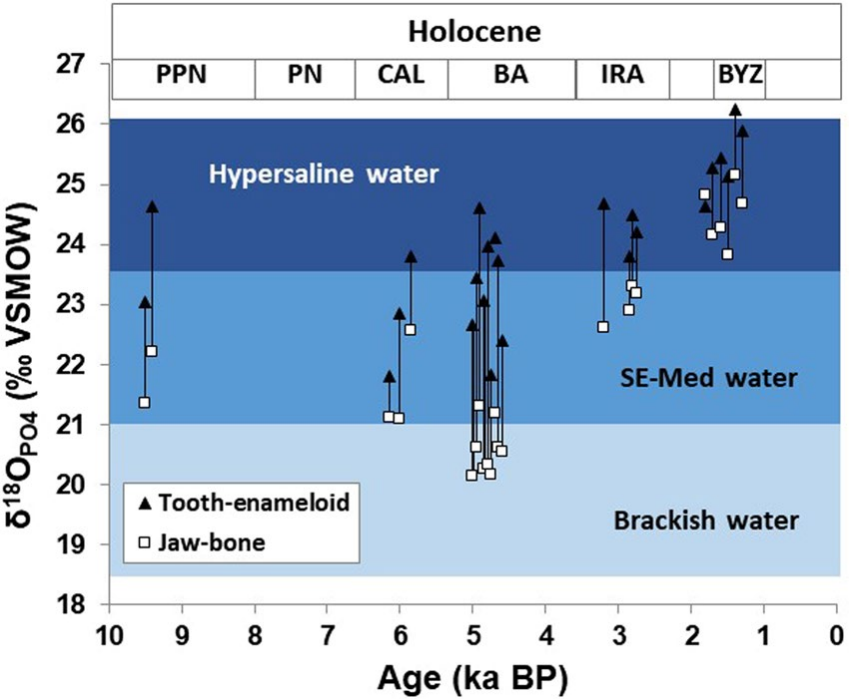
Comparison of δ^18^O_PO4_ values from teeth-jaw pairs of *S*. *aurata* individuals from different archaeological time periods. Pre-Pottery Neolithic: PPN; Pottery Neolithic: PN, Chalcolithic: CAL, Bronze Age: BA, Iron Age: IRA, and Byzantine period: BYZ. Note that for the Byzantine period both bone and enameloid δ^18^O_PO4_ values reflect hypersaline water, indicating that these fish lived their full life span in the Bardawil lagoon. Values lower than the southeast Mediterranean (southeast Mediterranean) range may reflect either fish migration into brackish lagoons or a certain degree of diagenetic alteration of the bone tissue in low δ^18^O_water_. Overall, δ^18^O_PO4_ values reflect the distinct salinity levels of the water bodies in which *S*. *aurata* mineralised their bones and teeth. The molariform tooth δ^18^O_PO4_ values thus indicate in which setting they were caught.

Mid-Holocene (Chalcolithic period and the Early Bronze Age: 4,500–2,500 years BCE; 6,500–4,500 years BP), δ^18^O_PO4_ values indicate that *S*. *aurata* was primarily captured ‘locally’, namely along the southeast Mediterranean coast (Table S1), however, data are insufficient for reconstructing the variability of fishing grounds for this time span. Currently, we have no data regarding the Middle Bronze Age (MBA). However, by the end of the LBA (~1,200 years BCE; ~3200 years BP), and onwards the fish-harvesting pattern in the southeast Mediterranean changed drastically, shifting to exploitation of *S*. *aurata* from a hypersaline source almost exclusively (1-Way ANOVA: F = 7.0978, p < 0.001; Figs 4A and 5). Whereas only 13.9% of the teeth (*n* = 33) analysed from pre-LBA contexts (PPN to EBA) had δ^18^O_PO4_ values indicative of hypersaline habitats, 84.2% of the teeth (*n* = 57) from the LBA to the Iron Age (IA) were characterised by high δ^18^O_PO4_ values typical for fish from hypersaline habitats. This pattern also prevails in the Byzantine period (n = 10). The δ^18^O_PO4_ values of sparid teeth from the LBA onwards are similar to those of extant *S*. *aurata* from the Bardawil lagoon (Figs 3 and 4A). In addition, from the late LBA onwards, both dentary bone and teeth of the same specimens of *S*. *aurata* displayed hypersaline isotopic signatures, suggesting that those fish may have spent their entire life cycle in the Bardawil lagoon (Fig. 6).

The δ^18^O_PO4_ values of tooth-jaw pairs of ancient *S*. *aurata* specimens from PPN to Byzantine are positively correlated (*n* = 24, *r*^2^ = 0.68), yet δ^18^O_PO4_ values of jawbones are consistently lower than those of tooth enameloid. An average offset (Δ^18^O_tooth-jaw_) of 1.8 ± 0.95‰ may reflect systematic differences of bioapatite biomineralisation in bones and enameloid. These bone-tooth δ^18^O_PO4_ differences suggest that the bone δ^18^O signatures likely represents a mean of the entire lifespan of S. *aurata*. This further supports that S. *aurata* spent a significant part of their adult life in the open sea, but continued to exploit hypersaline coastal lagoons as part of their trophic migration, as is the case with modern *S*. *aurata*^11–13^.

Specimens from the Byzantine period have oxygen isotope signatures typical of hypersaline water both in their teeth and jawbone (Fig. 6). This suggests that these fish lived their full life cycle in the hypersaline Bardawil lagoon. Jawbones from other archaeological periods have lower δ^18^O_PO4_ values than the teeth from the same individual/jaw, reflecting a marine origin and indicating that these fish lived predominantly in southeast Mediterranean seawater. A few jawbones have values lower than those expected for bioapatite forming in isotope equilibrium with modern southeast Mediterranean seawater. Some alteration of bone, which is known to be more prone to diagenesis than enameloid^24^, in low δ^18^O meteoric water during burial may have shifted δ^18^O_PO4_ towards lower values.

## Fish Body Mass as Indicator for Fishing Intensity

The body size of *S*. *aurata* (i.e. total length-TL (cm) and body mass-BM (kg)), can be calculated from the maximum length of the molariform tooth crown^48^. Body size of *S*. *aurata* reflects ontogenetic age and can thus be used as a proxy of fish exploitation (Fig. S3). Smaller average fish body size in both archaeological and modern contexts is associated with higher intensity exploitation of their nursery, i.e. the Bardawil lagoon^11,13,29^. During the Holocene, the size pattern of *S*. *aurata* clearly changes, exhibiting a decrease and lower range in fish size (i.e. harvesting of younger individuals) for specimens from the hypersaline lagoonal waters of the Bardawil (Table S1; Figs 4B and 5). The values estimated for *S*. *aurata* body size (body mass and total length) show significant decrease in the range and average size. The range of *S*. *aurata* decreased from a mean BM of 1.6 ± 0.8 kg (*n* = 33) and TL of 45.0 ± 8.3 cm during PPN–Early Bronze Age (EBA) to a mean BM of 1.1 ± 0.4 kg (*n* = 57) and TL of 40.7 ± 5.1 during LBA–IA, and to a mean of 0.78 ± 0.4 kg (*n* = 10) and mean TL of 36.4 ± 3.8 in the Byzantine period (Figs 4B and 5), approaching the average fish size of modern aquafarm *Sparus* (~0.45 kg) (for BM: 1-Way ANOVA: F = 3.9968; p < 0.0001; F = 3.532; For TL: p < 0.001; F = 3.267).

This trend of reduction in fish body size accords chronologically well with cultural changes and spreads over a millennial time-scale, starting in the LBA, continuing to the Iron Age and to the Byzantine period. Interestingly, we witness the appearance of larger fish only at Tel Dor, where we identified in the past *S*. *aurata* captured from the Mediterranean littoral zone^28^. Overall, the reduction in *S*. *aurata* mean and maximum body size demonstrates similarity with present day fishery data from the Bardawil lagoon (age group 1–3 years), where *S*. *aurata* are intensively exploited due to their abundance in this unique hypersaline nursery^11,13^. The decrease in *S*. *aurata* body size, to a range similar to present-day fish from the lagoon^11,13,40^ is in line with observations from traditional extensive aquaculture^49,50^.

From the LBA onwards we find evidence for extensive and likely year-round exploitation of *S*. *aurata* in the Bardawil lagoon. This unique exploitation pattern continues until the present^10,13^, and agrees well with the definition of traditional extensive aquaculture^49,50^. It is similar, for example, to the Italian ‘vallicoltura’ or the Egyptian ‘hosha’ – both representing traditional fish exploitation systems which utilise natural fish traps by taking advantage of the trophic migration of juveniles from the sea into coastal lagoons. These systems therefore capitalise on naturally occurring foods from this highly productive lagoon^11–13^. *Sparus aurata* were traditionally exploited extensively in coastal lagoons, until intensive rearing systems for this fish were developed during the 1980s^51^. Therefore, the cradle of marine aquaculture may be rooted in the Bardawil hypersaline lagoon. For more than 2,000 years it functioned as the major source for *Sparus aurata* for the Levant. This started not later than the LBA (~1,200 years BCE; 3200 years BP) as extensive harvesting and still continues today as intensive aquafarming^11,13^.

## A Diachronic Summary: Fish Exploitation in The Context of Egypto-Levantine Exchanges

In the southern Levant, for the last 50,000 years, *S*. *aurata* were exploited by local coastal fishing communities^4,5^. The first evidence for fishing in hypersaline lagoons appears during the PPNB ca. 9,500 years BP, but these remains are scarce and are insufficient to indicate systematic exploitation of hypersaline lagoons during this period. However, the occurrence of fish remains in Neolithic inland sites of the Judean Mountains (central Israel) indicates that the transportation of dry fish from the Mediterranean coast was already established in the Early Holocene^4,52^.

From the LBA period onwards, non-local (“exotic”) *S*. *aurata* with hypersaline δ^18^O_PO4_ signatures were imported from the Bardawil lagoon, almost entirely replacing *S*. *aurata* caught locally in coastal waters. These results contradict the conventional null hypothesis assuming that in coastal sites of the Levant *S*. *aurata* remains will represent local fishing, exhibiting that regardless of the site location (coastal or inland) most of the *S*. *aurata* were “exotic” (nonlocal) with δ^18^O_PO4_ signatures of the Bardawil lagoon.

In archaeological sites across the southern Levant, this fundamental change in fish provenance coincides with a sharp increase in the abundance of exotic Nilotic fish, such as Nile perch (*Lates niloticus*) and the Nile catfish^6,7^ (Table S3). Our results, therefore, provide new evidence for the intensity of Egypto-Canaanite long-distance, inter-regional trade connections in this period, which seem to have even included commercialization of fish from Bardawil lagoon. This pattern, which further intensified in the Iron Age and lasted at least until the Byzantine period, most likely comprised dried *S*. *aurata*, as depicted in Egyptian reliefs^53^ and as observed from the fragmentation patterns on some of the *S*. *aurata* remains recovered in Jerusalem (inland)^54^.

## Contextualising the Results Historically

Prior to Classical times, the densest network of archaeological sites in northern Sinai is documented for the LBA, the imperial epoch of Egypt’s New Kingdom. In this extremely arid, inhospitable region, meaningful population and economic infrastructure could only be sustained when backed by a centralised power. During the LBA Egypt controlled Canaan^55^ and well-documented terrestrial and maritime routes across northern Sinai and along the coast served as the main military and commercial artery between the two regions. Intensive traffic between them is recorded both textually and archaeologically, the latter including dozens of waystations, granaries, reservoirs, etc.^56,57^. The Egyptians called this route The Ways of Horus—the southernmost leg of the famed international trunk road linking Egypt with the Fertile Crescent, better known today as the Via Maris.

It is easy to envisage how the Bardawil fish industry and export emerged and functioned in this context. Although our earliest substantial evidence of *S*. *aurata* teeth with Bardawil-like, hypersaline δ^18^O_PO4_ signatures in Canaan dates to the early 12^th^ century BCE (at Lachish, an Egyptian administrative centre), the single 14^th^ century BCE hypersaline specimen found at Tel Rehov may hint at an earlier beginning of this trade during the LBA. Moreover, although we lack data from the MBA (~2,000–1,500 BCE), it is possible that this phenomenon started even earlier. Throughout the MBA, contact between Canaan and Egypt was close, although at the end of this period administrative control was inversed: Canaanites controlled parts of Egypt at this time. Egypto-Canaanite commerce flourished in this period too, though most of it appears to have been conducted via the sea^56^. Dozens of MBA settlements have been surveyed in northern Sinai, especially south of the Bardawil lagoon.

Importantly, our findings demonstrate that despite climatic changes and frequent socio-political, economic and demographic upheavals in both regions, once industry and marketing were in motion, they lasted at least until Byzantine times (i.e. minimally for two millennia), providing a paradigmatic example of a Mediterranean exchange network driven by the diversity and interdependence between ecological micro-regions^58,59^.

As if to support our main claim in this paper, Rabbi Abbahu, a Jewish sage living in 4^th^-century CE Caesarea Maritima—a major Southeast Mediterranean harbour city (just south of Tel Taninim, Fig. 2)—declared that: “any fish [brought to the city] must come either from Apamea [in Syria] or from Pelusium [Bardawil’s harbour town from the 6^th^ century BCE until the drying up of the eastern arm of the Nile^60^]”. Our results support Safrai’s concomitant assumption that even in coastal markets most of the fish in Roman Palestine were imported and demonstrate that this state of affairs had already been in place many centuries earlier.

## Methods

A sample of 100* S*. *aurata* molariform teeth recovered from 12 archaeological sites in the southern Levant was analysed in this study. Identification to species level is based on modern Mediterranean ichthyofauna housed at the University of Haifa reference collection and on OL’s personal research collection. Modern *S*. *aurata* teeth for this study were obtained from specimens captured offshore in Haifa Bay, Israel^28^. Their phosphate oxygen isotope signatures as well as those of extant *S*. *aurata* from the Bardawil lagoon^23^ were compared with those of teeth from archaeological *S*. *aurata* to assess their past habitats, specifically whether they derive from southeast Mediterranean marine or hypersaline lagoonal waters.

### Analytical Methods

The enamel cap (~0.2–0.4 mm thickness) of each tooth was separated from the dentine using a diamond-head micro-dental drill, washed three times with distilled water, and dried overnight at 50 °C. Each sample was crushed and ground to powder using an agate mortar and pestle. Organic matter was removed from the samples soaking the samples in 2% NaOCl overnight. The phosphate fraction of the samples was separated using a method modified after Dettmann *et al*.^61^ and described in detail by Gehler *et al*.^62^. In summary, approximately 5 mg of pretreated sample powder was dissolved in 0.8 ml 2 M HF and placed on a vibrating table for ca. 12 h. After centrifuging, the supernatant sample solution was separated from the CaF_2_ precipitate and transferred to new centrifuge tubes. After neutralising the HF solution with NH_4_OH (25%) in the presence of bromothymol blue as a pH indicator, Ag_3_PO_4_ was rapidly precipitated by adding 0.8 ml of 2 M AgNO_3_. Following settling of the Ag_3_PO_4_ crystals, the samples were centrifuged and the supernatant solution was removed using a pipette. The Ag_3_PO_4_ was then rinsed five times with MilliQ water and dried overnight in an oven at 50 °C.

Ag_3_PO_4_ aliquots of 0.5 mg were placed into silver capsules and analysed in triplicate by means of high temperature reduction using a Finnigan TC-EA coupled via a Conflo III to a Micromass 100 GC-IRMS at the University of Mainz, or to a Finnigan Delta Plus XL GC-IRMS at the Universities of Tübingen and Lausanne, following Vennemann *et al*.^63^. Measured ^18^O/^16^O isotope ratios are reported in the δ-notation:

$${{\rm{\delta }}}^{{\rm{18}}}{{\rm{O}}}_{{\rm{sample}}}=[({}^{{\rm{18}}}{\rm{O}}/{}^{{\rm{16}}}{{\rm{O}}}_{{\rm{sample}}}-{}^{{\rm{18}}}{\rm{O}}/{}^{{\rm{16}}}{{\rm{O}}}_{{\rm{VSMOW}}})/{}^{{\rm{18}}}{\rm{O}}/{}^{{\rm{16}}}{{\rm{O}}}_{{\rm{VSMOW}}}-1]\times 1000$$

i.e., as the deviation in per mil (‰) relative to Vienna Standard Mean Ocean Water (VSMOW), the international reference material. The δ^18^O_PO4_ values were measured with an external precision of ±0.3‰ (1 SD).

The raw δ^18^O_PO4_ values were normalised to an Ag_3_PO_4_ standard produced by Elemental Microanalysis with a certified value of 21.7‰ (silver phosphate P/N IVA33802207, batch no. 180097, distributed by IVA Analysentechnik, Germany). The analytical precision for this standard was better than ±0.3‰ (1σ). For untreated NIST SRM 120c Florida phosphate rock standard reference material, we obtained a δ^18^O_PO4_ value of 21.9 ± 0.3‰ (n = 9). This value compares well with the values around 21.7‰ initially measured by Lécuyer *et al*.^64^ and currently reported by most other laboratories as compiled in Chenery *et al*.^65^.

### The δ^18^O_PO4_ theoretical range of Sparidae bioapatite

We calculated the equilibrium range of δ^18^O_PO4_ in the littoral of the southeast Mediterranean and in hypersaline lagoons, evolving from typical southeast Mediterranean water. The calculation is based on the temperature-dependent relation for isotope fractionation during biomineralisation of apatite by Lécuyer *et al*.^22^: T °C = 117.4–4.5(δ^18^O_PO4_ − δ^18^O_SeaWater_), where δ^18^O_PO4_ and δ^18^O_SeaWater_ correspond to the isotope compositions of bio-apatite and seawater relative to VSMOW, respectively. This relation is valid for the temperature range of 8 °C < T < 32 °С relevant for the water temperatures encountered in the Mediterranean realm^37,38^.

### Body mass estimation of *S*. *aurata*

Body mass of ancient fish can be estimated from species-specific regressions with bone or molariform tooth size. In this study, we used the tooth length measurements recommended for the first molariform tooth (Fig. S1), and regression equations to estimated fish total length (cm) and body mass (kg)^48^:

Linear regression calculated from *S*. *aurata* first molariform tooth maximum length (FMTL) to fish total length (TL).

[2] TL = 32.21*FMTL + 154.07 (r2 = 0.83; Fig. S3)

Linear regression demonstrating the correlation between TL to fish body mass (FBM)

[3] FBM = 1.7086*e^−05^*TL^2.977^ (r^2^ = 0.98).

## Electronic supplementary material

Supplementary Information
