# Supplementary material for: Tooth oxygen isotopes reveal Late Bronze Age origin of Mediterranean fish aquaculture and trade
Source: Sci Rep. 2018 Sep 20;8:14086. doi: 10.1038/s41598-018-32468-1 (PMC6148281; doi:10.1038/s41598-018-32468-1)
Supplement: Supplementary file 1 — Supplementary Information [file 41598_2018_32468_MOESM1_ESM.docx]

**Supporting Information**

**Tooth oxygen isotopes reveal Late Bronze Age origin of Mediterranean fish aquaculture and trade**

Sisma-Ventura Guy^a,b,c^*, Tütken Thomas^b^*, Zohar Irit^d,e^, Pack Andreas^c^*, Sivan Dorit^f,g^, Lernau Omri^e^, Gilboa Ayelet^e, h^, Bar-Oz Guy^e, h^*

a. Israel Oceanographic & Limnological Research, Haifa, Israel

b. Institute for Geosciences, Johannes-Gutenberg-University of Mainz, Mainz, Germany

c. Department of Isotope Geology, Georg-August-University of Göttingen, Göttingen, Gemany

d. Oranim Academic College, Kiryat Tivon, Israel

e. Zinman Institute of Archaeology, University of Haifa, Haifa, Israel

f. Department of Maritime Civilizations, Charney School of Marine Sciences

g. The Leon Recanati Institute for Maritime Studies, University of Haifa, Haifa, Israel

h. The Department of Archaeology, University of Haifa, Haifa, Israel

*Corresponding authors: [guy.siv@ocean.org.il](mailto:guy.siv@ocean.org.il) (G. Sisma-Ventura); [tuetken@uni-mainz.de](mailto:tuetken@uni-mainz.de) (T. Tütken); [Andreas.Pack@geo.uni-goettingen.de](mailto:Andreas.Pack@geo.uni-goettingen.de) (A. Pack); [guybar@research.haifa.ac.il](mailto:guybar@research.haifa.ac.il) (G. Bar-Oz)

**Dating of *S. aurata* remains from archaeological layers**

Archaeological periodisation in the Levant is mostly based on the seriation and stylistic development of the most common artifacts: flint objects in prehistoric times and mainly ceramic vessels from the Pottery Neolithic and onwards. These are pegged into an absolute (calendrical) time scale either by directly dating finds by chemical/physical means, the most commonly-used being ^14^C dating, or, for historical periods only, by historical dating of assemblages of artifacts or entire habitation levels. This too can be direct (such as by the occurrence of artifacts inscribed with the name of historically-known kings, or coins with well dated inscriptions), but also indirect—for example the presumed (and thus less certain) identification of an archaeological phenomenon (e.g., a destruction level) with a historically-known event. The dating of a single context/settlement is usually not based on data originating only from the specific site in question, but also takes into consideration information from all other relevant contemporaneous sites. After more than a century of extensive excavation in the southern Levant the general periodisation and chronology of the archaeological periods is well established and accepted, though uncertainties and concomitant debates continue at the margins, which are occasionally wide for certain archaeological periods^1,2^ (Table **S2**). For the fish specimens investigated in this study, no direct dating exists, neither for the items themselves, nor for the specific deposits (loci) in which they were found. Therefore, in Supplementary data, Table **S1** (the fifth column) we list the archaeological periods of the general strata in which they were found. The absolute dates given there (in brackets) usually follow the dates proposed by the excavators/researchers of the sites. They either comprise the whole range of the archaeological period in question, or, often—in cases where a more specific date could be pinpointed—a shortened age range within that period. The exception to this is the Iron Age, a relatively recent period from which most of the analysed specimens derive. The absolute dates of this period and especially its sub-periods are very significant historically since they overlap with crucial historical phenomena such as the rise of territorial states and kingship in the Levant. These dates have been a matter of controversy over the last decades, resulting in extensive radiometric dating at several sites^3,4,5,6^. This dating has narrowed the debate to ± 50 years, which is very close to the method’s limit of detection, and currently the discussion seems to be at an impasse. Consequently, the absolute dates for Iron Age items in supplementary data Table **S1** represent the entire chronological range that in our opinion is currently valid within the range constrained by ^14^C dating in the Levant.

1. Sharon, I. Chapter 4: Levantine Chronology. In A. E. Killebrew and M. Steiner (eds.). *Oxford Handbook of the Archaeology of the Levant (ca. 8000–332 BCE*). Oxford University Press, pp. 44–68 (2014).

2. Stern, E. & Gilboa, A. (eds.). *The New Encyclopedia of Archaeological Excavations in the Holy Land* I–IV. Jerusalem and New York: Israel Exploration Society and Simon and Schuster (1993).

3. Sharon, I., Gilboa, A., Jull, A. J. T. & Boaretto, E. Report on the First Stage of the Iron Age Dating Project in Israel: Supporting a Low Chronology. *Radiocarbon* **49**, 1–46 (2007).

4. Mazar, A. & Bronks Ramsey, C. A. Response to Finkelstein and Piasetzky’s Criticism and ‘New Perspective’. *Radiocarbon* **52**, 1681–1688 (2010).

5. Finkelstein, I. The Iron Age chronology debate: is the gap narrowing? *Near Eastern Archaeology* **74**, 50–54 (2011).

6. Toffolo, M. B., Arie, E., Martin, M. A. S., Boaretto, E. & Finkelstein I. Absolute Chronology of Megiddo, Israel, in the Late Bronze and Iron Ages: High-Resolution Radiocarbon Dating. *Radiocarbon* **56**, 221–244 (2014).

**Fig. S1**. Anatomic location of *Sparus aurata* dentary and the first molariform teeth in the jaw after Sisma-Ventura et al. (2015)^28^. The length of the first molariform tooth was used to estimate fish body mass and length.


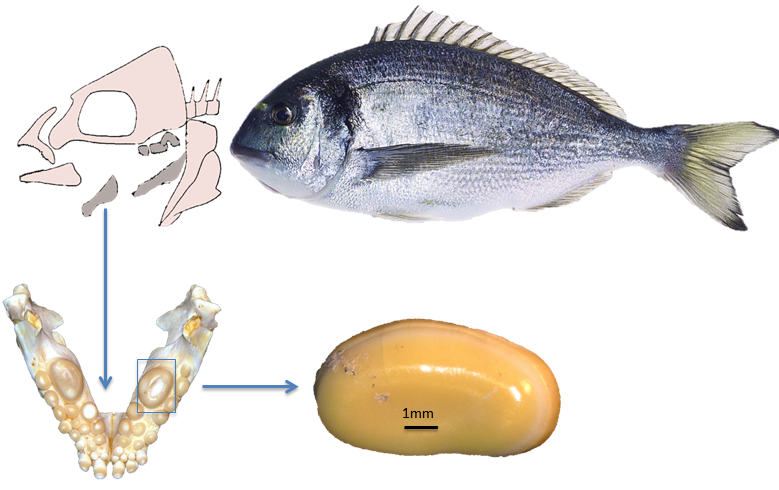


**Fig. S2.** δ^18^O_SW_ and salinity relationship of the Mediterranean surface water: former measurements of the Western and Eastern Mediterranean, taken during 1988-1989 [blue diamonds and blue circles] and during 2009-2010 [blue triangles]; data compilation from Sisma-Ventura et al. (2016)^37^. The data of the Bardawil lagoon are plotted as dark blue squares^31^.


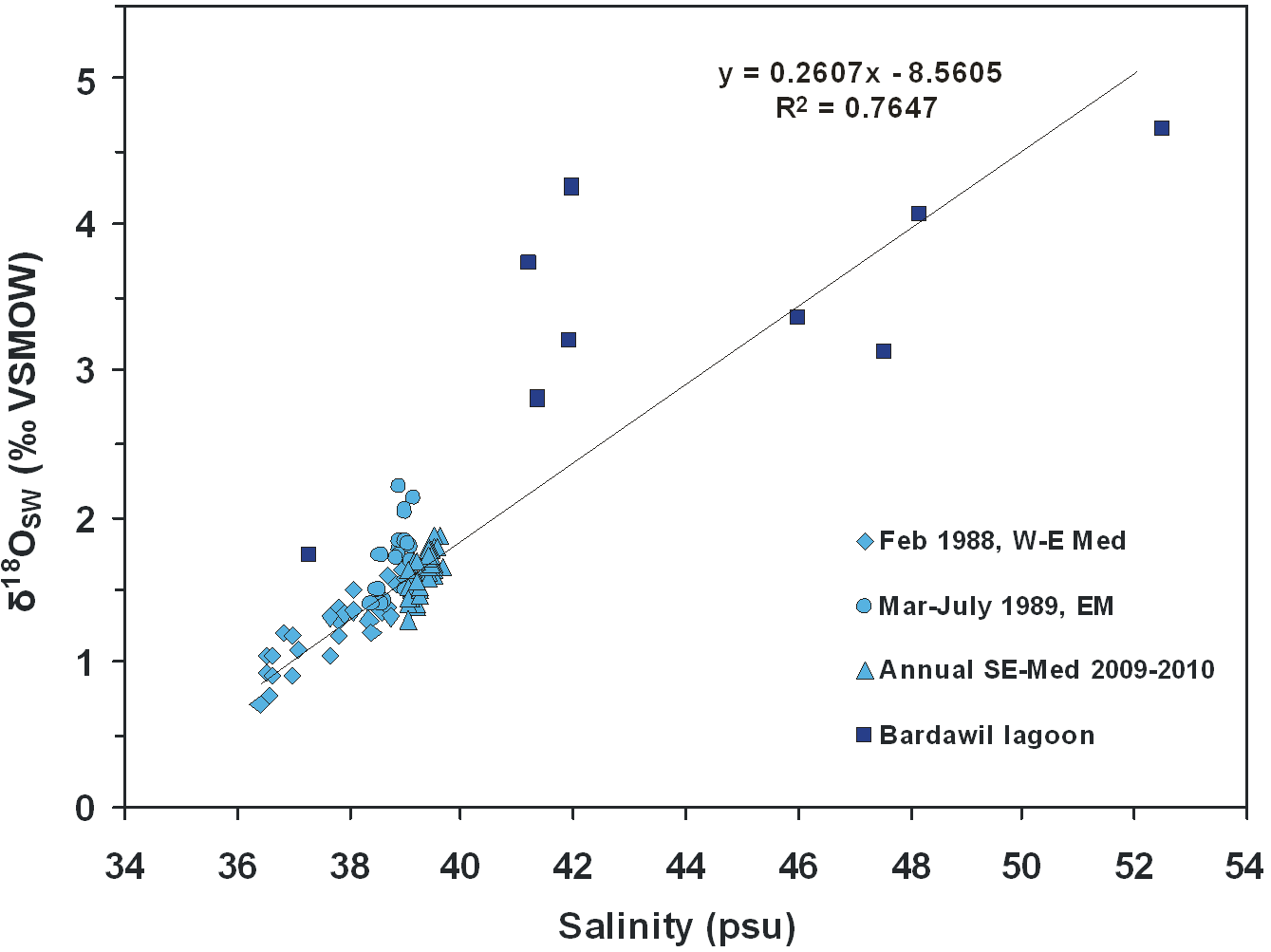


**Fig. S3:** Linear regression calculated for body size estimation (total length = TL) of *S. aurata,* from the first molariform tooth maximum length (modified after Desse and Desse-Berset, 1996)^48^. Six modern samples from the Southeast Med littoral were added in this study.


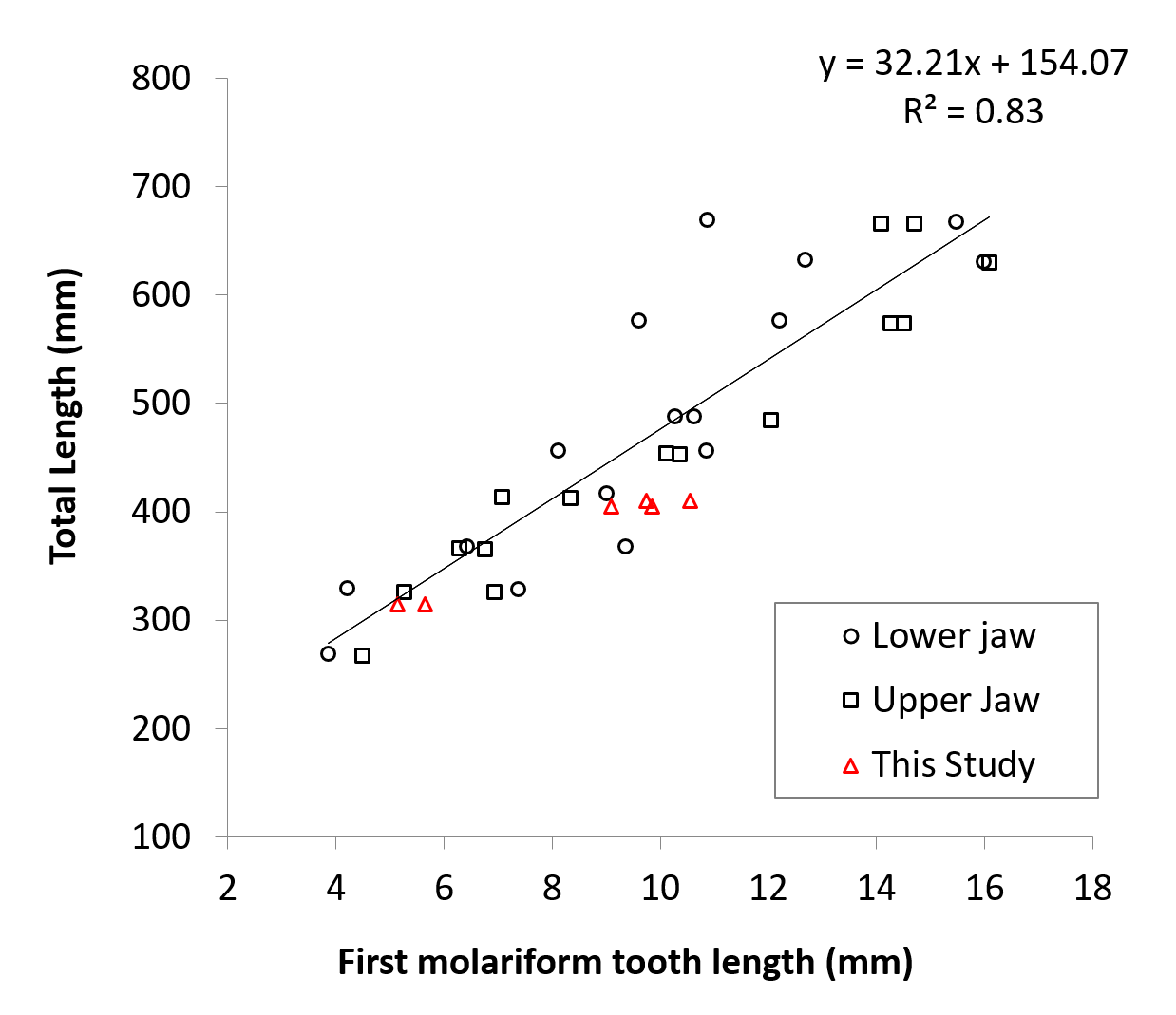


**Table S1:** *Sparus aurata* teeth used in this study, in chronological order^1^, δ^18^O_PO4_ values and molariform tooth length as markers for water salinity level and fish body size (Total length-TL and Body mass-BM), respectively.

| **Site** | **Sample** | **CAT#** | **Context** | **Archaeological Period** | **Tooth** | **Salinity** | **δ^18^O_PO4_** | | **TL** | **BM** |
| --- | --- | --- | --- | --- | --- | --- | --- | --- | --- | --- |
|  |  |  | **(locus, stratum)** |  | **Length [mm]** | **level** | **[‰ VSMOW]** | | **[cm]** | **KG** |
| **Hatoula** | Tooth | 00183 | Square F; Lzz71 | PPNA (9,750-8,500 BCE) ^2^ | 4.10 | M | | 22.0 | 28.61 | 0.37 |
|  | Tooth | 00215 | Square F; L71 |  | 10.6 | M | | 21.9 | 49.55 | 1.90 |
|  | Tooth | 00221 | Square F; Lb70 |  | 9.50 | M | | 23.4 | 46.01 | 1.52 |
|  | Tooth | 00225 | Square F; Lzz70 |  | 5.70 | M | | 23.1 | 33.77 | 0.61 |
|  | Tooth | 00316 | Square G; L== |  | 7.10 | M | | 22.5 | 38.28 | 0.88 |
|  | Tooth | 00232 | Square F; La69 |  | 11.2 | M | | 22.7 | 51.49 | 2.13 |
|  | Tooth | 00297 | Square G; L == |  | 10.2 | M | | 21.4 | 48.26 | 1.76 |
| **Hatoula** | Tooth | 00829 | Square F; Lb70 | PPNB (8,500-7,000 BCE) ^2^ | 12.3 | S | | 24.1 | 55.03 | 2.60 |
|  | Tooth | 00757 | Square F; Ld66 |  | 8.40 | S | | 25.4 | 42.47 | 1.20 |
|  | Tooth | 00590 | Square F; Lzz71 |  | 9.40 | M | | 22.4 | 45.69 | 1.49 |
|  | Tooth | 00533 | Square F; Lc72 |  | 6.30 | S | | 24.9 | 35.70 | 0.72 |
|  | Tooth | 00467 | Square F; L67 |  | 4.30 | M | | 21.4 | 29.26 | 0.40 |
|  | Tooth | 00537 | Square F; Ld67 |  | 8.20 | M | | 23.0 | 41.82 | 1.15 |
|  | Bone |  |  |  |  | M | | 21.4 |  |  |
|  | Tooth | 00572 | Square F; Le69 |  | 9.70 | M | | 23.2 | 46.65 | 1.59 |
|  | Tooth | 00704 | Square F; Le66 |  | 11.3 | S | | 24.6 | 51.81 | 2.17 |
|  | Bone |  |  |  |  | M | | 22.2 |  |  |
|  | Tooth | 00929 | Square F; Ld73 |  | 10.2 | M | | 23.4 | 48.26 | 1.76 |
|  | Tooth | 00933 | Square F; Ld73 |  | 5.8 | M | | 23.2 | 34.09 | 0.62 |
|  | Tooth | 00472 | Square F; L63 |  | 9.7 | S | | 25.2 | 46.65 | 1.59 |
| **Ashkelon-Afridar** | Tooth | 00095 | Basket 03913 | PPNC (7,000-6,500 BCE)^3^ | 11.7 | M | | 22.8 | 53.10 | 2.34 |
|  | Tooth | 00158 | Basket 04254 |  | 5.90 | S | | 26.4 | 34.41 | 0.64 |
|  | Tooth | 00267 | Basket 04382 |  | 14.6 | M | | 23.0 | 62.44 | 3.78 |
|  | Tooth | 00275 | Basket 04192 |  | 7.10 | M | | 21.1 | 38.28 | 0.88 |
|  | Tooth | 00301 | Basket 01090 |  | 9.70 | M | | 22.6 | 46.65 | 1.59 |
| **Gilat** | Tooth | 00023 | Square H2; L961 | Chalcolithic (5,500-3,900/3,700 BCE)^4^ | 5.30 | M | | 21.6 | 32.48 | 0.54 |
|  | Bone |  |  |  |  | M | | 21.1 |  |  |
|  | Tooth | 00035 | Square B5; L16 |  | 12.6 | M/S | | 23.8 | 56.00 | 2.74 |
|  | Bone |  |  |  |  | M | | 22.6 |  |  |
|  | Tooth | 00023 | Square H2; L961 |  | 10.3 | M | | 22.8 | 48.59 | 1.79 |
|  | Bone |  |  |  |  | M | | 21.1 |  |  |
| **Ashkelon-Afridar** | Tooth | 00013 | L34 | EB I (3,800- 3,400BCE)^5^ | 11.9 | M | | 22.7 | 53.74 | 2.42 |
|  | Bone |  |  |  |  | B | | 20.1 |  |  |
|  | Tooth | 00013 | L34 |  | 8.20 | M | | 23.1 | 41.82 | 1.15 |
|  | Bone |  |  |  |  | B | | 20.3 |  |  |
| **Ashkelon - Barnea** | Tooth |  |  | EB I (3,550-3,000 BCE)^5^ | 10.8 | M | | 22.4 | 50.20 | 1.98 |
|  | Tooth |  |  |  | 8.90 | M | | 23.4 | 44.08 | 1.34 |
|  | Tooth |  |  |  | 9.60 | S | | 24.6 | 46.33 | 1.56 |
|  | Tooth |  |  |  | 12.7 | M | | 21.8 | 56.32 | 2.78 |
|  | Tooth |  |  |  | 9.40 | M/S | | 23.7 | 45.69 | 1.49 |
| **Lachish** | Tooth | 00997 | Area S, L3938, Str VI | LB III (first half of 12^th^ c. BCE) ^6^ | 6.70 | S | | 24.8 | 36.99 | 0.80 |
|  | Tooth | 01093 | Area S , L3918, Str VI |  | 8.20 | S | | 25.6 | 41.82 | 1.15 |
|  | Tooth | 01165 | Area S, L3927, Str VI |  | 8.20 | M/S | | 23.5 | 41.82 | 1.15 |
|  | Tooth | 00226 | Area S, L3701, Str VI |  | 6.90 | S | | 25.1 | 37.63 | 0.84 |
|  | Tooth | 00229 | Area S, L3701, Str VI |  | 7.40 | S | | 24.7 | 39.24 | 0.95 |
|  | Tooth | 00237 | Area S, L3579, Str IV | IA II (late 9^th^/ 8^th^ c. BCE) ^6^ | 7.30 | S | | 24.1 | 38.92 | 0.93 |
|  | Tooth | 00238 | Area S, L3643, Str IVb |  | 6.20 | M/S | | 23.9 | 35.38 | 0.70 |
|  | Tooth | 00227 | Area S, L670, Str IVc |  | 7.70 | S | | 25.3 | 40.21 | 1.02 |
|  | Tooth | 00199 | Area S, L3643, Str IVb |  | 9.70 | S | | 24.3 | 46.65 | 1.59 |
|  | Tooth | 00200 | Area S, L3670, Str IVc |  | 8.20 | S | | 24.2 | 41.82 | 1.15 |
| **Tel Rehov** | Tooth | 01043 | Area D; L7951; local Str D9b | LB IIA (late 14^th^ c. BCE) ^7^ | 8.00 | S | | 25.1 | 41.18 | 1.10 |
|  | Tooth | 00999 | Area C, L8465; Str V | Late IA IIA (late 10^th^/ 9^th^ c. BCE) | 9.20 | S | | 24.7 | 45.04 | 1.43 |
| **Ashkelon** | Tooth | 00571 | Grid 38; Square 64; Layer S | Early IA I (second half of 12^th^ c. BCE) ^8,9^ | 6.00 | S | | 24.8 | 34.73 | 0.66 |
|  | Tooth | 01439 | Grid 38; Square 64; Layer S | Early IA I (second half of 12^th^ c. BCE) | 8.60 | S | | 25.5 | 43.11 | 1.26 |
|  | Tooth | 01439 | Grid 38; Square 64; Layer S | Early IA I (second half of 12^th^ c. BCE) | 8.30 | S | | 25.1 | 42.14 | 1.17 |
|  | Tooth | 01562 | Grid 38; Square 64; Layer 149 | Early IA I (second half of 12^th^ c. BCE) | 10.6 | S | | 26.1 | 49.55 | 1.90 |
|  | Tooth | 01440 | Grid 38; Square 64; Layer S | Early IA I (second half of 12^th^ c. BCE) | 9.00 | S | | 24.5 | 44.40 | 1.37 |
|  | Tooth | 01438 | Grid 38; Square 64; Layer S | Early IA I (second half of 12^th^ c. BCE) | 8.80 | S | | 25.0 | 43.75 | 1.31 |
|  | Tooth | 06192 |  | IA I (12^th^ c. BCE) | 10.0 | S | | 26.1 | 47.62 | 1.69 |
|  | Tooth | 06381 |  | IA I (12^th^-early 10^th^ c. BCE) | 7.70 | S | | 25.4 | 40.21 | 1.02 |
|  | Tooth | 06184 |  |  | 6.50 | M/S | | 23.9 | 36.35 | 0.76 |
|  | Tooth | 07246 |  |  | 6.70 | S | | 24.3 | 36.99 | 0.80 |
|  | Tooth | 06218 |  |  | 5.00 | M/S | | 23.8 | 31.51 | 0.49 |
|  | Tooth | 06429 |  |  | 7.40 | S | | 24.2 | 39.24 | 0.95 |
|  | Tooth | 06460 |  |  | 9.10 | S | | 25.6 | 44.72 | 1.40 |
|  | Tooth | 06739 |  |  | 6.60 | S | | 26.0 | 36.67 | 0.78 |
|  | Tooth | 06241 |  |  | 4.20 | S | | 24.6 | 28.94 | 0.38 |
|  | Tooth | 06289 |  |  | 4.60 | S | | 25.3 | 30.22 | 0.44 |
|  | Tooth | 06380 |  |  | 6.30 | S | | 25.1 | 35.70 | 0.72 |
| **Tel Dor** | Tooth |  | Area D2/Phase13, L08D2-262 | IA I (mid-12^th^ -mid 11^th^ c. BCE)^10,11^ | 6.59 | M | | 21.6 | 36.64 | 0.77 |
|  | Tooth |  | Area D1/Phase 10, L05D1-541 | Early IA IIA (late-10^th^-mid-9^th^ c. BCE) | 10.9 | M | | 22.3 | 50.58 | 2.02 |
|  | Tooth |  | Area D5/Phase 10, L06D5-049 |  | 10.3 | M | | 23.0 | 48.72 | 1.81 |
|  | Tooth |  | Area D2/Phase pre-7, L07D2-068 | IA II (late-10^th^-early 8^th^ c. BCE) | 9.53 | S | | 25.2 | 46.11 | 1.53 |
|  | Tooth |  | Area D2/Phase pre-7, L07D2-069 |  | 8.92 | M | | 21.7 | 44.14 | 1.35 |
|  | Tooth |  | Area D2/Phase pre-7, L07D2-017 |  | 7.35 | S | | 23.7 | 39.08 | 0.94 |
|  | Tooth |  | Area D2/Phase pre-7, L07D2-017 |  | 9.53 | M | | 22.7 | 46.11 | 1.53 |
|  | Tooth |  | Area D2/Phase pre-7, L09D2-324 |  | 10.2 | S | | 24.6 | 48.17 | 1.75 |
|  | Tooth |  | Area D2/Phase pre-7, L09D2-324 |  | 9.53 | M | | 22.5 | 51.65 | 2.15 |
|  | Tooth |  | Area D5/Phase 6a, L05D2-802 | Iron II (late 8^th^ -mid-7^th^ c. BCE) | 8.62 | M/S | | 23.5 | 43.17 | 1.26 |
|  | Tooth |  | Area D5/Phase 6a, L05D2-802 |  | 8.42 | S | | 25.2 | 42.53 | 1.21 |
| **Tel Miqne** | Tooth | 01063 | Str Vb; 4NW8.302 | IA I (11^th^/early 10^th^ c. BCE) ^12,13^ | 6.70 | S | | 24.7 | 36.99 | 0.80 |
|  | Tooth | 01063 | Str Vb; 4NW8.302 |  | 6.70 | S | | 26.1 | 36.99 | 0.80 |
|  | Tooth | 01063 | Str Vb; 4NW8.302 |  | 5.20 | S | | 25.1 | 32.16 | 0.52 |
|  | Tooth | 01063 | Str Vb; 4NW8.302 |  | 6.10 | S | | 25.2 | 35.06 | 0.68 |
|  | Tooth | 01063 |  |  | 7.20 | S | | 25.7 | 38.60 | 0.90 |
|  | Tooth | 00280 | Str IVa; 4NW24.151 | IA II (10^th^/9^th^ c. BCE) | 5.80 | S | | 24.3 | 34.09 | 0.62 |
|  | Tooth | 01266 | Str IVa; 4NW24.139 |  | 6.40 | S | | 24.9 | 36.02 | 0.74 |
| **Jerusalem pool** | Tooth | 01000 |  | IA II (late 9^th^/early 8^th^ c. BCE) ^14^ | 7.50 | S | | 24.8 | 39.57 | 0.97 |
|  | Tooth | 01001 |  |  | 8.20 | S | | 26.0 | 41.82 | 1.15 |
|  | Tooth | 01002 |  |  | 7.30 | S | | 25.1 | 38.92 | 0.93 |
|  | Tooth | 01003 |  |  | 9.30 | S | | 24.5 | 45.37 | 1.46 |
|  | Tooth | 01004 |  |  | 8.20 | M/S | | 23.8 | 41.82 | 1.15 |
|  | Tooth | 01005 |  |  | 10.0 | S | | 24.5 | 47.62 | 1.69 |
|  | Tooth | 01006 |  |  | 7.80 | S | | 25.4 | 40.53 | 1.05 |
|  | Tooth | 01007 |  |  | 9.40 | S | | 24.3 | 45.69 | 1.49 |
|  | Tooth | 01008 |  |  | 8.40 | S | | 24.5 | 42.47 | 1.20 |
|  | Tooth | 01011 |  |  | 7.10 | M/S | | 23.9 | 38.28 | 0.88 |
| **Tel Taninim** | Tooth | 0418 | Square A8; L1303; Basket 1004 | Byzantine (4^th^-7^th^ c. CE) ^15^ | 9.10 | M | | 22.7 | 44.72 | 1.40 |
|  | Tooth | 00057 | Square A8; L1303;Basket 1006 | | 6.70 | M/S | | 23.8 | 36.99 | 0.8 |
| **Halutsa** | Tooth |  | Square 4/04-C, Basket 39 | Byzantine (6^th^ c. CE) ^16^ | 6.40 | S | | 24.6 | 36.02 | 0.74 |
|  | Bone |  |  |  |  | S | | 24.8 |  |  |
|  | Tooth |  | Area A, Basket 27 |  | 5.80 | S | | 25.1 | 34.09 | 0.62 |
|  | Bone |  |  |  |  | M/S | | 23.8 |  |  |
|  | Tooth |  | Square 4/07-2, Basket 72 |  | 7.10 | S | | 25.3 | 38.28 | 0.88 |
|  | Bone |  |  |  |  | S | | 24.2 |  |  |
| **Shivta** | Tooth |  | Area K, Basket 1693 | Byzantine (6^th^ c. CE) ^17^ | 4.60 | S | | 25.4 | 30.22 | 0.44 |
|  | Bone |  |  |  |  | S | | 24.3 |  |  |
|  | Tooth |  |  |  | 5.60 | S | | 24.8 | 33.45 | 0.59 |
|  | Tooth |  | Area A, Basket 3 |  | 6.30 | S | | 25.6 | 35.70 | 0.72 |
|  | Bone |  |  |  |  | S | | 24.7 |  |  |
| **Tamra** | Tooth | 00162 |  | Early Islamic (early 8^th^ c. CE) ^18^ | 6.40 | S | | 25.9 | 36.02 | 0.74 |
|  | Tooth | 00155 |  |  | 7.30 | S | | 26.2 | 38.92 | 0.93 |
|  | Bone |  |  |  |  | S | | 25.2 |  |  |

**M = marine, B = brackish, BCE = Before Common Era; c = century; IA – Iron Age; L = locus; EBA = Early Bronze Age; LBA = Late Bronze Age; S = hypersaline; Str = stratum**

^1^ Registration numbers are in different formats since they follow those assigned by the different excavators. Chronological resolution is determined by the nature of the contexts in which the bones and teeth were found.

^2^ Lechevallier, M., Avraham, R., and Patricia, C. Anderson. 1994. Le gisement de Hatoula en Judée occidentale, Israël: rapport des fouilles 1980-1988. No. 8. Association Paléorient.

^3^ Garfinkel,Y., Dag, D., Hesse, B., Wapnish, P., Rookis, D., Hartman, G., et al. 2005. Neolithic Ashkelon: Meat Processing and Early Pastoralism on the Mediterranean Coast. *Eurasian Prehistory*, **3**, 43–72.

^4^ Levy, T.E. (ed.) .2006. *Archaeology, Anthropology and Cult*. *The Sanctuary at Gilat, Israel*. London, Equinox.

^5^ Golani A. 2013. The transition from the Late Chalcolithic to the Early Bronze I in southwestern Canaan – Ashkelon as a case for continuity. *Paleorient* **39,** 95–110.

^6^ Ussishkin, D. 2004. *The Renewed Archaeological Excavations at Lachish (1973–1994)*. 5 Vols. Tel Aviv: Tel Aviv Institute of Archaeology Monograph Series 22.

^7^ <http://www.rehov.org/Rehov/stratigraphic.htm>

^8^ Stager, L.E., Schloen, J.D. and Master, D.M. 2008. *Ashkelon 1*. Introduction and Overview (1985-2006). [Final Reports of the Leon Levy Expedition to Ashkelon.](javascript:ViewSeries(%22Final%20Reports%20of%20The%20Leon%20Levy%20Expedition%20to%20Ashkelon%20-ASHK%22)) [Winona Lake. Harvard Semitic Museum and Eisenbrauns](javascript:ViewPublisher(%22Harvard%20Semitic%20Museum%20/%20Eisenbrauns%22)).

^9^ Master, D.M, Stager, L.E., and Yasur-Landau, A. 2011. Chronological Observations at the Dawn of the Iron Age in Ashkelon,” Ägypten und Levante **21**, 261–280.

^10^ Sharon, I., and Gilboa, A. 2013. The *ŠKL* Town: Dor in the Early Iron Age. In A. E. Killebrew and G. Lehmann (eds.). *The Philistines and Other “Sea Peoples” in Text and Archaeology.* The Society of Biblical Literature: Atlanta, pp. 393–468.

^11^ Gilboa, A., Sharon, I., and Bloch-Smith, E. 2015. Capital of Solomon’s Fourth District? Israelite Dor. *Levant* **47**/1, 51–74.

^12^ Dothan, T., and Zukerman, A. 2004. A Preliminary Study of the Mycenaean IIIC: 1Pottery Assemblages from Tel Miqne-Ekron and Ashdod. *Bulletin of the American Schools of Oriental Research* **333**, 7–61.

^13^ Ben-Shlomo, D., Shai, I., Maeir, A.M. 2004. Late Philistine Decorated Ware ("Ashdod Ware"): Typology, Chronology, and Production Centers. *Bulletin of the American Schools of Oriental Research* **335**, 1–35.

^14^ [De Groot](http://www.tandfonline.com/author/de+Groot%2C+Alon), A. and [Fadida](http://www.tandfonline.com/author/Fadida%2C+Atalya), A. 2011. The Pottery Assemblage from the Rock Cut Pool near the Gihon Spring. *Tel Aviv* **38**/2, 158–166.

^15^ Hirschfeld, Y. and Birger-Calderon, R. 1991. Early Roman and Byzantine Estates near Caesarea. *Israel Exploration Journal* **41**, 81–111.

^16^ Bar-Oz, G., Weissbrod, L. and Erickson-Gini, T. Haluza. 2016. A preliminary report. *Excavations and surveys in Israel* **128** (2016).

^17^ Tepper, Y., Erickson-Gini, T., Farhi, Y. and Bar-Oz, G. 2017. Shivta, in light of the Renewed Excavations: Preliminary report of the 2015-2017 seasons. *Tel Aviv.*

^18^ Di Segni, L. and Tepper, Y. 2004. A Greek inscription dated by the era of Hegira in an Umayyad church at Tamra in eastern Galilee. *Liber Annuus* **54**, 343–350.

**Table S2:** The absolute chronology of the archaeological periods in the southern Levant following Sharon (2014) and Stern and Gilboa (1993).

| **Archaeological period** | **Years** |
| --- | --- |
| Neolithic (Pre Pottery - PPN) | 9,750 - 6,500 BCE |
| Neolithic (Pottery - PN) | 6,500 - 4,500 BCE |
| Chalcolithic (CAL) | 4,500 - 3,900/3,700 BCE |
| Early Bronze Age (EBA) | 3,900/3,700 - 2,500/2,300 BCE |
| Intermediate Bronze Age (IBA) | 2,500/2,300 - 2,200/1,900 BCE |
| Middle Bronze Age (MBA) | 2,200/1,900 - 1,650/1,540 BCE |
| Late Bronze Age (LBA) | 1,650/1,540 - 1,200/1,150 BCE |
| Iron Age (IA) | 1,200/1,150 - 600 BCE |
| Babylonian | 600 - 530 BCE |
| Persian | 530 - 332 BCE |
| Hellenistic | 332 - 63 BCE |
| Roman | 63 BCE - 324 CE |
| Byzantine (BYZ) | 324 - 638 CE |
| Early Islamic | 638 - 1,099 CE |

**Table S3:** Abundances (%) of identified fish remain from archaeological sites in Israel as function of their habitat, presented in chronological order.

| **Period** | **Site** | **Marine** | **Freshwater** | **Nile** |
| --- | --- | --- | --- | --- |
| Natufian | El Wad^1^ | 100 |  |  |
| Natufian | Hatoula^2,3^ | 100 |  |  |
| PPN | Hatoula | 99.7 | 0.3 |  |
| PPNC | Atlit Yam^4^ | 100 |  |  |
| PPNC | Ashkelon^5^ | 99.7 | 0.3 |  |
| Pottery Neolithic | Ziqim^6^ | 87.0 |  | 13.0 |
| Pottery Neolithic | Neve Yam^7^ | 100 |  |  |
| Late Chalcolithic | Namir Road^8^ | 98.9 | 1.1 |  |
| Early Bronze Age | Ashkelon-Afridar^9^ | 97.9 |  | 1.9 |
| Early Bronze Age I | Tel Qashish^10^ | 40.0 | 60.0 |  |
| Mid Bronze Age II | City of David^11^ | 66.7 | 11.1 | 22.2 |
| Late Bronze Age III | Lachish^12^ | 64.5 | 6.1 | 29.4 |
| Late Bronze Age III | Haruvit^13^ | 95.4 | 4.1 | 0.4 |
| Late Bronze Age II | Tel Dor-Area G 11-12^14^ | 25.7 |  | 74.3 |
| Late Bronze Age | Tel Harasim^15^ | 58.3 |  | 41.7 |
| Late Bronze Age | Timna^16,17^ | 97.9 | 2.1 |  |
| Late Bronze Age II | Tel Aphek^18^ | 87.5 |  | 12.5 |
| Late Bronze Age -Iron Age II | Tel Yoqneam^19^ | 35.1 | 8.1 | 56.8 |
| Early-Iron Age | Tel Dor Area D^20^ | 47.8 | 0.5 | 51.6 |
| Iron Age | Tel Dor-Area G 10^20^ | 88.6 | 1.8 | 9.6 |
| Iron Age | *el* Ahwat^21^ | 25.5 | 2.0 | 72.5 |
| Iron Age II | Tel Aphek^22^ | 40.0 | 20.0 | 40.0 |
| Iron Age II | Tel Harasim^23^ | 61.1 |  | 38.9 |
| Iron Age II | Jerusalem-pool^24^ | 75.1 | 17.5 | 7.5 |
| Iron Age II | City of David^11^ | 72.1 | 12.0 | 15.8 |
| Iron Age II | Jerusalem-Area G^11^ | 69.8 | 28.9 | 1.3 |
| Iron Age II | Jerusalem-Haophel^25^ | 68.1 | 14.9 | 17.0 |
| Roman | Caesarea Cespit 3898^26^ | 89.0 | 11.0 |  |
| Early Roman-Byzantine | Ein Gedi^27^ | 51.5 | 48.5 |  |
| Persian, Helenistic, Byzantine | City of David^11^ | 47.1 |  | 52.9 |
| Persian, Byzantine | Tel Harasim^28^ | 57.1 |  | 42.9 |
| Early Byzantine | Upper Zohar^29^ | 84.8 | 0.6 | 14.6 |

^1^ Valla F.R., Bar-Yosef O., Smith P., Tchernov E., Desse J. 1986. Un noveau sondage sur la terrace d'El Ouad, Israel, Paléorient 12, 21–38.

^2^ Davis S.J.M. 1985. A preliminary report of fauna from Hatula: a Natufian-Khiamian (PPNA) site near Latroun, Israel, in: Lechevallier, M., Ronen, A. (Eds.), Le site Natufian-Khiamien de Hatoula, pres de Latroun, Israel., Centré de Rechérché Français de Jerusalem, Jerusalem, pp. 71–118.

^3^ Lernau H., Lernau, O. 1994. The Fish Remains, in: Lechevallier, M., Ronen, A. (Eds.), Le Gisement de Hatoula en Judee occidentale, Israel., Association Plaeorient, Memoires et Travaux du Centre de Recherche Francais de Jerusalem, Paris, pp. 111–121.

^4^ Galili E., Lernau O., Zohar I. 2004. Fishing and coastal adaptations at 'Atlit-Yam- A submerged PNC fishing village off the Carmel Coast, Israel, Atiqot, 1–34.

^5^ Lernau O. 2008. The fish bones, in: Garfinkel, Y., Dag, D. (Eds.), Neolithic Ashkelon, Qedem Monographs of the Institute of Aerchaeology, The Hebrew University, Jerusalem, pp. 263–268.

^6^ Garfinkel Y., Dag D., Horwitz L.K., Lernau O., Mienis H.K. 2002. Ziqim, A Pottery Neolithic site in the southern coastal plainof Israel-A final report, Mitekufat Ha'even, Journal of the Israel Prehistoric Society 32, 73–146.

^7^ Horwitz L.K., Lernau O., Galili E. 2006. Fauna from the submerged Pottery Neolithic sote of Newe yam, Northern israel, Journal of The Israel Prehistoric Society 36, 139-171.

^8^ Van den Brink E.C.M., et al., 2016. Late Chalcolithic settlement remains East of Namir Road., Journal of Israel Prehistoric Society 46, 20–121.

^9^ Lernau O. 2004. Fish remains from Early Bronze Age Ashqelon, Afridar, Atiqot 45, 299–303.

^10^ Horwitz L.K. 2003. Fauna from Tel Qashish, in: Ben-Tor, A., Bonfil, R., Zuckerman, S. (Eds.), Tel Qashish: A Village in The Jezreel Valley. Final Report of The Archaeological Excavations (1978-1987), Qedem Reports, The Institute of Archaeology, the Hebrew University of Jerusalem, Jerusalem, pp. 427–443.

^11^ Lernau H., Lernau O., 1992. Fish Remains, in: De Groot, A., Ariel, D.T. (Eds.), Excavations at the city of David 1978-1985: Final report.Vol. III., The Institute of Archaeology, The Hebrew University, Jerusalem., Jerusalem, pp. 131–148.

^12^ Lernau O., Golani D. 2004. Section B: The osteological remains (aquatic), in: U., U. (Ed.), The Renewed Archaeological excavations at Lachish (1973-1994), Emery and Claire Yass Publications in Archaeology, Tel Aviv, pp. 2456–2489.

^13^ Van Neer W., Zohar I., Lernau O. 2005. The emergence of fishing communities in the eastern Mediterranean region: A survey of evidence from pre- and protohistoric periods, Paléorient 31, 131–157.

^14^ Bartosiewicz L., Zohar I., Lisk E. in press. Non-Mammalian Vertebrate Remains, in: Gilboa, A., Sharon, I., Zorn, J.R. (Eds.), Excavations at Dor, Final Report. Volume II. Area G: The Late Bronze and Iron Ages, Qedem Reports, Hebrew University, Jerusalem, pp. 1293–1316.

^15^ Lernau O. 2000. Fish remains at Tel Harassim, in: Givon, S. (Ed.), The Sixth Season of Excavation at Tel Harassim (Nahel Barkai) 2000, Bar-Ilan University, Tel-Aviv University, pp. 4–13.

^16^ Lernau H.,1988. Fish Remains from two smelting camps and the Timna temple, in: Rothenberg, B. (Ed.), The Egyptian Minning temple at Timna., London, pp. 241–246.

^17^ Van Neer, W. et al., 2004. Fish remains from archaeological sites as indicators of former trade connections in the Eastern Mediterranean, Paléorient 30, 101–148.

^18^ Lernau O. 2009. Fish Remains, in: Gadot, Y., Yadin, E. (Eds.), Aphek-Anthpatris II, Monograph Series Of The Sonia And Marco Nadler Institute Of Archaeology, Tel Aviv, Israel, pp. 569–574.

^19^ Horwitz L.K., Bar Giora N., Mienis H.K., Lernau O. 2005. Faunal and malacoogical remains from the Middle Bronze. Late Bronze and Iron Age levels at Tel Yoqne'am, in: Ben-Tor, A., D., B.-A., Livneh, A. (Eds.), Yone'am II: The Middle and Late Bronze Ages. Final report of the Archaeological Excavations (1977-1988), Qedem Reports, Jerusalem, pp. 395–431.

^20^ Raban-Gerstel N., Bar-Oz G., Zohar I., Sharon I., Gilboa A. 2008. Early Iron Age Dor (Israel): A faunal perspective, Bulletin of The American School of oriental Research 349, 25–59.

^21^ Lernau O. 2011. Fish remains from *el*-Ahwat, in: Zertal, A. (Ed.), El-Ahwat: A Fortified Site from the Early Iron Age Near Nahal 'Iron, Israel. Excavations 1993-2000, Brill, Leiden, pp. 362–368.

^22^ Lernau O. 2009. Fish Remains, in: Gadot, Y., Yadin, E. (Eds.), Aphek-Anthpatris II, Monograph Series Of The Sonia And Marco Nadler Institute Of Archaeology, Tel Aviv, Israel, pp. 569–574.

^23^ Lernau O. 2000. Fish remains at Tel Harassim, in: Givon, S. (Ed.), The Sixth Season of Excavation at Tel Harassim (Nahel Barkai) 2000, Bar-Ilan University, Tel-Aviv University, pp. 4–13.

^24^ Reich R., Shukron E., Lernau O. 2008. Recent discoveries in the City of david, Jerusalem, Israel Exploration Journal 57, 153–169.

^25^ Lernau H., Lernau O. 1989. Fish bone remains, in: Mazar, B., Mazar, E. (Eds.), Excavation in the south of the temple mount: The Ophel of the Biblical Jerusalem., Qedem, Jerusalem, pp. 155–161.

^26^ Lernau O. 2015. Fish Bones from a Cesspit adjacent to Herod’s Circus, in: Porath, Y., Bijovsky, G., Cotton, H.M., Eck, W., Finkielsztejn, G., Gendelman, P., Gersht, R., Gorin-Rosen, Y., Gur, K., Katsnelson, N. (Eds.), Caesarea Maritima I: Herod’s circus and related buildings Part 2: The finds Israel Antiquities Authority, Jerusalem, pp. 203–216.

^27^ Lernau O. 2005. Fish remains from 'Ein Gedi, Atiqot 49, 49–56.

^28^ Lernau O. 2000. Fish remains at Tel Harassim, in: Givon, S. (Ed.), The Sixth Season of Excavation at Tel Harassim (Nahel Barkai) 2000, Bar-Ilan University, Tel-Aviv University, pp. 4–13.

^29^ Lernau O. 1995. The fish remains of Upper Zohar, in: Harper, R.P. (Ed.), Upper Zohar. An Early Buzantine Fort in Palestina Tertia. Final Report of Excavations in 1985-1986, Oxford University Press, Oxford, UK, pp. 99–161.
